# New keratinolytic bacteria in valorization of chicken feather waste

**DOI:** 10.1186/s13568-018-0538-y

**Published:** 2018-01-24

**Authors:** Wojciech Łaba, Barbara Żarowska, Dorota Chorążyk, Anna Pudło, Michał Piegza, Anna Kancelista, Wiesław Kopeć

**Affiliations:** 1 Department of Biotechnology and Food Microbiology, Wrocław University of Environmental and Life Sciences, Chełmońskiego 37, 51-630 Wrocław, Poland; 2 Department of Animal Products Technology and Quality Management, Wrocław University of Environmental and Life Sciences, Chełmońskiego 37, 51-630 Wrocław, Poland

**Keywords:** Keratinase, *Kocuria rhizophila*, Feathers, Optimization, Biodegradation

## Abstract

There is an increasing demand for cost-effective and ecologically-friendly methods for valorization of poultry feather waste, in which keratinolytic bacteria present a great potential. Feather-degrading bacteria were isolated from living poultry and a single strain, identified as *Kocuria rhizophila* p3-3, exhibited significant keratinolytic properties. The bacterial strain effectively degraded up to 52% of chicken feathers during 4 days of culture at 25 °C. Zymographic analysis revealed the presence of two dominating proteolytic enzymes in the culture fluid. Culture conditions were optimized in order to maximize the liberation of soluble proteins and free amino acids. A two-step procedure was used, comprising a Plackett–Burman screening design, followed by a Box–Behnken design. Concentration of feather substrate, MgSO_4_ and KH_2_PO_4_ were the most influential parameters for the accumulation of soluble proteins in culture *K. rhizophila* p3-3, while feathers and MgSO_4_ also affected the concentration of amino acids. The resultant raw hydrolysate supernatant, prior to and after additional treatments, was rich in phenylalanine, histidine, arginine and aspartic acid. Additionally the hydrolysate exhibited radical-scavenging activity and ferric reducing power.

**Electronic supplementary material:**

The online version of this article (10.1186/s13568-018-0538-y) contains supplementary material, which is available to authorized users.

## Introduction

Intense development of human economic activity, including agricultural and animal production, as well as leather processing industries is associated with the discharge of by-products into the environment. Despite the fact that the amount of waste animal tissues from poultry industry is relatively low as compared with processing of other animal products, waste management of hardly degradable keratin, mainly feathers, poses significant difficulties (Kopec et al. 2014). The annual global waste of chicken feathers is at 8.5 million tons (Fellahi et al. 2014). Feathers are composed of 95–98% protein, predominantly β-keratin. The dominating amino acids in its structure comprise: cysteine, glutamine, proline, as well as serine, the most abundant amino acid (Tiwary and Gupta 2012). Keratins are insoluble in water and exhibit high resistance to physical and chemical treatments, as well as typical proteolytic enzymes. The degradation of these proteins is possible with the participation of specific microbial proteolytic enzymes—keratinases, frequently supported by chemical or enzymatic reducing agents (Lange et al. 2016).

Typical techniques for keratin waste processing into feed ingredients include mechanical, hydrothermal and thermo-chemical treatments, to facilitate protein digestion and assimilability. However, these modifications are usually costly and energy-consuming, and the resulting products in large part are characterized by low nutrition value, volatility of the amino acid composition, as well as deficiency in basic amino acids (Coward-Kelly et al. 2006; Staron et al. 2010). Additional treatments with concentrated alkalies (KOH, NaOH, Ca(OH)_2_) or reducing compounds (Na_2_SO_3_, Na_2_S), despite increased efficiency of keratin hydrolysis lead to the formation of another troublesome effluents, loss in methionine, lysine and tryptophan, followed by formation of non-protein amino acids, lanthionine, lysoalanine (Gupta et al. 2013).

Since severe legal restrictions have been put in 2000 among the European Union on the application of processed animal tissues for feeding livestock, the demand on keratin meals undergoes a significant decline (Korniłłowicz-Kowalska and Bohacz 2011). This is the reason for the increasing interest in novel routes for the management over increasing input of keratinous waste stream.

As biotechnological methods are considered as cost-effective and environment-friendly, an interesting alternative to these techniques is microbial degradation, due to the lower cost, mild process conditions, lack of the ecological hazard and the output of potentially relevant products. Microorganisms break down keratin to peptides and amino acids, that accumulate in culture medium, and are partially metabolized as basic building elements—carbon and nitrogen (Vasileva-Tonkova et al. 2009). The interest in microbiologically obtained keratin hydrolysates is driven by a variety of their prospective applications. Another route for bioconversion of keratin waste is hydrolysis with cell-free keratinase extracts or purified keratinases. This approach allows for more controlled hydrolysis. Moreover, when combined with thermal or thermo-chemical pretreatment, it becomes applicable in production of hydrolysates with advantageous amino acid balance, at high efficiency.

Keratinases and the follow-on keratin hydrolysates may also be applied in obtaining cheap, useful products, such as nitrogen-rich fertilizers, compostable films, biodegradable materials and reinforced fabrics (Singh and Kushwaha 2015). Keratinases could be effective as a components of detergents, in manufacturing of personal care products and modification of fibers, such as wool or silk. Their prospective applications also their use in medicine for the treatment of psoriasis and acne, as an adjunct in the nails diseases treatment, as well as in prion proteins degradation (Gupta and Ramnani 2006; Selvam and Vishnupriya 2012). Moreover, keratin hydrolysis products may be considered as a potential source of bioactive peptides (Choinska et al. 2011). Recently, peptides of various biological activity have been described, after obtaining through microbial fermentation of chicken feathers. Among them, peptides of anti-oxidative potential are of special attention, due to the growing interest in applicable natural antioxidants (Fakhfakh et al. 2011; Fontoura et al. 2014).

Nevertheless, other applications of keratinases should be denoted as exceptionally promising in industrial circumstances. One of the target areas is leather industry, where keratinases support or carry out the dehairing process, allowing to at least partially replace lime-sulfide treatment. Also, application of keratin hydrolysates allowed for the reduction of chromium effluents from the process of tanning (Balaji et al. 2008). Another vital area is the introduction of keratinolytic microorganisms the initial biodegradation stage, preceding the bioconversion keratin hydrolysates into biogas (Patinvoh et al. 2016).

Numerous bacteria, actinomyces and filamentous fungi, including dermatophytic species, have been described as keratin decomposers. The dominant group of microorganisms capable of keratinases biosynthesis are bacteria of the genus *Bacillus*: among others, *B. subtilis*, *B. pumilus*, *B cereus*, *B. coagulans*, *B. licheniformis* or *B. megatherium*. Degradation of keratin proteins can also be conducted by a number of other Gram-positive bacteria *Lysobacter*, *Nesternokia*, *Kocuria*, *Microbacterium*, and some Gram-negative bacteria, e.g. *Vibrio*, *Xanthomonas*, *Stenotrophomonas* and *Chryseobacterium*. Similar abilities were found among microorganisms thermo- and extremophilic, representatives by types: *Fervidobacterium*, *Thermoanaerobacter*, *Nesternokia*, *Bacillus* (Nam et al. 2002; Gupta and Ramnani 2006; Brandelli et al. 2015).

Here we describe the isolation and screening of keratinolytic bacteria that effectively decompose chicken feathers, as well as optimization of culture conditions for one bacterial isolate to maximize accumulation of proteins and amino acids and characterization of the resultant hydrolysate.

## Materials and methods

### Isolation

Microbiological material was obtained from domestic birds: chicken (*Gallus gallus*), goose (*Anser anser*), turkey (*Meleagris gallopavo*) and duck (*Cairina moschata*). Isolation of bacterial strains was performed with two methods: swab samples from 1 cm^2^ skin surface were washed with 0.1% Tween 80 and by washing 0.1 g feather samples for 30 min under agitation. The obtained suspensions were inoculated onto LB Agar and incubated for 72 h at 25 °C. The resultant colonies were collected, passaged and the isolates were screened for proteolytic activity.

### Screening of proteolytic isolates

Screening for proteolytic activity of isolates was performed in two stages. At first, each isolate was inoculated on skim milk agar (skim milk powder 8%, agar 2%) and incubated for 48 h at 25 °C, in order to determine the ratio (Q) between the clear zone around colonies and colony diameter expressed in millimeters. Afterwards, selected isolates with the highest Q were cultured in liquid medium (FM) composed of (% w/v): MgSO_4_ 0.1, KH_2_PO_4_ 0.01, FeSO_4_·7H_2_O 0.001, CaCl_2_ 0.01, yeast extract 0.05 and white chicken feathers (washed and degreased) 1.0. Cultures were carried out for 4 days, at 25 °C under 180 rpm agitation. Maximum values of soluble protein, free amino groups, reduced thiols and proteolytic activity of each isolate were compared. The most effective feather-degrading isolate, selected for further study, was deposited in the Polish Collection of Microorganisms (PCM) of the Institute of Immunology and Experimental Therapy Polish Academy of Sciences under Accession Number PCM 2931.

### Identification and molecular phylogenetic studies

The identification of selected bacterial isolates was based on the sequence analysis of the 16S rDNA genes. The product was amplified by the PCR with following universal primers: (27 F) AGAGTTTGATCGTGGCTCAG and (1492l R) GGTTACCTTGTTACGACT under standard procedure. The PCR product was purified from reaction components and sequenced using the same primers. The obtained sequences were subjected to Ribosomal Database Project (RDP) release 10 in order to find related nucleotide sequences. The sequence alignment and phylogenetic study was performed using MAFFT version 6 and Archaeopteryx version 0.9914 (Cole et al. 2014). The nucleotide sequences submitted to the GenBank database of the National Centre for Biotechnology Information (NCBI) under accession numbers listed in Table 2.

### Optimization of feather degradation by a selected bacterial isolate

Biodegradation of chicken feathers by a selected bacterial isolate was optimized using three-step methodology: selection of culture temperature, determination of significant factors affecting the process and optimization of three most influential parameters. The release of soluble proteins and amino acids from feathers during bacterial cultures served as measures of substrate biodegradation (dependent variables). Each value of dependent variables was a maximum outcome observed during 4-day cultures. All cultures were carried out in 250 mL conical flasks, in 50 mL of media.

The effect of culture temperature on the maximum level of soluble protein, free amino groups, proteolytic activity and substrate loss was evaluated in FM medium at 25–40 °C with 5 °C interval, under 180 rpm agitation.

Preliminary screening of factors affecting biodegradation of feathers was performed according to a Plackett–Burman factorial design. Seven factors were selected for the screening: concentration of feathers (A), MgSO_4_·7H_2_O (B), KH_2_PO_4_ (C), CaCl_2_ (D), yeast extract (E), quantity of inoculum (F) and agitation speed (G), used at two different levels coded as − 1 and + 1 (Table 1).

**Table 1:** Independent variables for the performed experimental designs in coded and natural values

Variable	Unit	Designation	(− 1)	(0)	(+ 1)
Plackett–Burman design					
Feathers	% (w/v)	X_1_	0.5	–	2.0
MgSO_4_·7H_2_O	% (w/v)	X_2_	0.05	–	0.20
KH_2_PO_4_	% (w/v)	X_3_	0.005	–	0.020
CaCl_2_	% (w/v)	X_4_	0.005	–	0.020
Yeast extract	% (w/v)	X_5_	0.02	–	0.10
Inoculum size	Cells/flask	X_6_	1.2 × 10^8^	–	1.2 × 10^9^
Agitation	rpm	X_7_	180	–	200
Box–Behnken design					
Feathers	% w/v	X_1_	1	3	5
MgSO_4_·7H_2_O	% w/v	X_2_	0.01	0.05	0.09
KH_2_PO_4_	% w/v	X_3_	0.01	0.05	0.09

Statistical optimization of three most influential parameters, concentration of feathers (A), MgSO_4_·7H_2_O (B) and KH_2_PO_4_ (C), was performed according to a 13-run Box–Behnken design with four replicates at the central point. Each culture run was performed in duplicate. Three levels of each independent variable were coded as − 1, 0 and + 1, according to Table 1. The relationship between the independent variables and the response was formulated as the second-order polynomial equation (Eq. 1):

1 $$\begin{aligned} {\text{Y}} \, & = \,\upbeta_{\text{0}} +\upbeta_{ 1} {\text{X}}_{ 1} +\upbeta_{ 2} {\text{X}}_{ 2} +\upbeta_{ 3} {\text{X}}_{ 3} +\upbeta_{ 1 1} {\text{X}}_{ 1} {\text{X}}_{ 1} +\upbeta_{ 2 2} {\text{X}}_{ 2} {\text{X}}_{ 2} \\ & \quad +\upbeta_{ 3 3} {\text{X}}_{ 3} {\text{X}}_{ 3} +\upbeta_{ 1 2} {\text{X}}_{ 1} {\text{X}}_{ 2} +\upbeta_{ 1 3} {\text{X}}_{ 1} {\text{X}}_{ 3} +\upbeta_{ 2 3} {\text{X}}_{ 2} {\text{X}}_{ 3} \\ \end{aligned}$$

where Y was the predicted response, β_0_ was the intercept and regression coefficients were designated as follows: β_1_, β_2_, β_3_ (linear), β_11_, β_22_, β_33_ (square) and β_12_, β_13_, β_23_ (interaction). The Box–Cox transformation, experimental design, polynomial equation fit, regression and ANOVA statistics, were performed with Statistica 12.5 software (StatSoft Inc.). Optimal values were obtained for the three dependent variables simultaneously using the Profiler tool of Statistica 12.5.

### Production and treatments of feather hydrolysate

Optimal culture conditions were adapted from the results of the Box–Behnken design to produce feather hydrolysate. After culture, the fluid was subjected to two methods of treatment: autoclaving (121 °C, 1 atm., 20 min) and sonification (5 min in cycles of 0.5 s/0.5 s, at 4 °C). The treated samples were centrifuged and a profile of free amino acids and antioxidative properties were determined for the supernatants.

### Analytical determinations

#### Proteolytic activity

Proteolytic activity was determined on bovine hemoglobin 1 mg/mL (Sigma-Aldrich), in Tris–HCl buffer pH 9.5 (0.05 M), at 55 °C. The reaction was terminated with trichloroacetic acid (TCA) 8%. The mixture was cooled for 20 min, centrifuged (12,000*g*, 10 min) and the absorbance was measured at the 280 nm wavelength. One unit of proteolytic activity was expressed as 1 μmol of released tyrosine calculated per 1 mL of culture fluid within 1 min.

#### Soluble proteins

Concentration of soluble proteins in culture fluids was determined using the Coomassie (Bradford) Protein Assay Kit (Thermo Scientific), with bovine serum albumin as a standard.

#### Amino acids

Concentration of free amino groups in culture fluids was determined with the ninhydrin method, with glycine as a standard (Sun et al. 2006).

#### Sulfhydryl groups

Concentration of reduced sulfhydryl groups was determined with Ellman’s reagent according to Riener et al. (2002).

#### Feather substrate loss

Substrate loss was determined after separation of the residual substrate on Whatman grade 2 filter paper and drying at 105 °C. The result was expressed as the percent of the initial amount of feathers introduced into culture media, with the consideration of the initial substrates moisture.

#### Radical scavenging capability

Following methods were applied for measuring the total antioxidant capacity of hydrolysates: ABTS radicals reducing activity of was determined using Trolox-equivalent antioxidant capacity assay according to Re et al. (1999), where the inhibition of ABTS+ radicals was compared to Trolox standard and expressed as micromole Trolox per 100 mL of hydrolysate; DPPH radicals scavenging activity determination was conducted according to Jang et al. (2008), except that the ethanolic solution of DPPH was used as described by Milardovic et al. (2006), where the ability to scavenge the DPPH radicals was calculated from data obtained for Trolox standard and expressed as micromole Trolox per 100 mL of hydrolysate; FRAP (ferric reducing antioxidant power) was assayed according to Benzie and Strain (1996) and was expressed as μmol of Fe^2+^ in relation to 100 mL of the hydrolysate.

### Zymography

Zymographic analysis of the culture supernatant of the selected bacterial isolate was performed. The sample was mixed at 1:1 ratio with the sample buffer (Tris–HCl 0.32 M; pH 6.8; glycerol 48%; SDS 8%; bromophenol blue 0.06%). Sample in the amount of 5 or 10 μL was loaded onto 12% polyacrylamide gel (5% staking gel) containing 0.1% of copolymerized casein. PAGE Ruler prestained (Thermo Scientific) was used as a reference marker. Electrophoresis was performed at constant 18 mA, at 2 °C. Subsequently, the gel was washed twice with Triton-X 2.5%, once with the incubation buffer and incubated for 24 h at 28 °C in the same buffer (Tris–HCl 0.05 M, pH 7.5, containing CaCl_2_ 2 mM and NaN_3_ 0.02%). Proteolytic activity bands were visualized by staining with Coomassie Blue and decolorization with methanol: acetic acid: water (50:10:40).

### Microscopic observations

Visual examination of feathers decomposed within bacterial culture was performed using scanning electron microscopy (SEM) on a Hitachi S3400 microscope.

### Amino acid profile of feather hydrolysate

The profile of free amino acids in feather hydrolysates was determined with HPLC, as described by Henderson et al. (2000). Initial derivatization with *O*-phthalaldehyde was performed. The analysis made on a HPLC 1100 Series system (Agilent Technologies) equipped with the ZORBAX Eclipse-AAA column, 4.6 × 150 mm, 3.5 μm (Agilent Technologies).

## Results

### Isolation and screening of keratinolytic bacteria

Domestic birds plumage and skin surface was used as a convenient source of proteolytic bacteria, of potentially keratinolytic properties. As a result of the isolation procedure, a total number of 55 isolates of proteolytic bacteria was obtained from 36 original samples. Spot tests on skim milk agar revealed several isolates of outstanding proteolytic activity, exhibiting clear zones width around colonies within a range of 5.5 and 10.5 mm (Additional file 1: Figure S1). Eight of the isolates were selected for liquid cultures in medium with feathers as a main nutrient source, where products of substrate decomposition and proteolytic activity were determined. Significant diversity in the concentration of hydrolysis products was observed among the tested isolates.

**Fig. 1:**
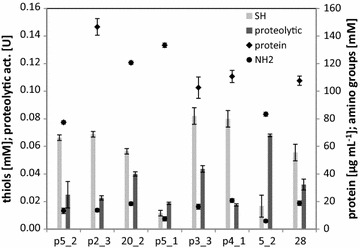
Comparison of keratinolytic potential of selected bacterial isolates grown in feather-containing medium. Maximum concentration of hydrolysis products and proteolytic activity from 4-day cultures was given. Light grey bars indicate concentration of reduced thiols; dark grey bars indicate proteolytic activity; diamonds indicate protein concentration; circles indicate concentration of amino acids

The concentration of soluble proteins ranged from 77 to 147 μg/mL and free amino acid groups from 5.82 to 20.74 mM (Fig. 1). In cultures of each of the tested isolates the presence of reduced thiols was confirmed, within a range of 0.012–0.082 mM. Likewise, in each case comparably moderate caseinolytic activity was observed, between 0.019 and 0.068 U. Nevertheless, none of the isolates prevailed in terms of each measured factor simultaneously. The isolate p3-3 was selected for further study, as exhibiting reasonable value of all tested parameters, especially high level of reduced thiols and amino acids.

### Identification of bacterial isolates

**Fig. 2:**
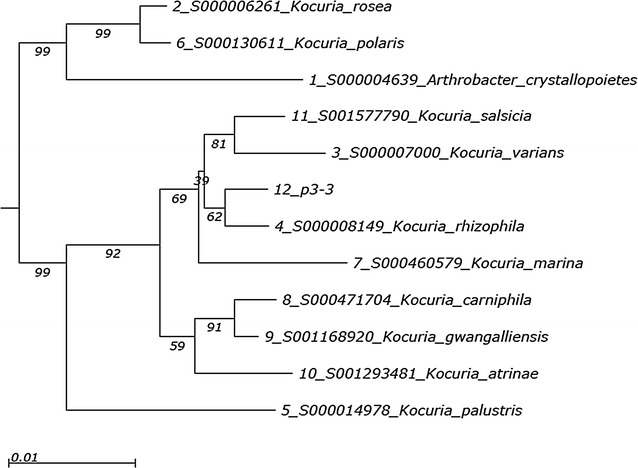
Phylogenetic tree indicating a position of the p3-3 isolate within *Kocuria* genus based on 16S rDNA. Phylogenetic tree was built with the neighbor-joining method from the relationships of 16S rDNA sequences between the isolate p3-3 and closely related type strains. Bootstrap values are indicated at the branching points (percent values from 500 replicate bootstrap samplings). The bar represents evolutionary distance of 0.01

The initial comparison of the 16S rDNA partial sequences of the nine tested isolates with the RDP database revealed that most of them belong to the *Kocuria* genus, where six isolated were identified as *K. rhizophila*, and a single strain of *Pantoea anthophila*, with high identity score (Table 2). The neighbor joining phylogenetic tree demonstrated the location of the strain p3-3 in the branch comprising *K. varians*, *K. salsicia* and *K. marina*, specifically on the sub-branch of *K. rhizophila* (Fig. 2).

**Table 2:** Identification results for the selected feather-degrading bacterial isolates

Isolate designation	Identification result	S_ab score^a^	GenBank accession number^b^
p5-2	*Kocuria rhizophila*	0.962	MG230492
p2-3	*Kocuria rhizophila*	0.961	MG230434
20-2	*Kocuria rhizophila*	0.966	MG230441
p5-1	*Kocuria rhizophila*	0.965	MG230442
p3-3	*Kocuria rhizophila*	0.967	MG230324
p4-1	*Kocuria rhizophila*	0.959	MG230493
5–2	*Pantoea anthophila*	0.959	MG230494
28	*Kocuria rhizophila*	0.961	MG230529

^a^Maximum “seqmatch score” calculated by RDP database SeqMatch tool

^b^Accession numbers of 16S rDNA sequences submitted to NCBI GenBank database

### Degradation of feathers in cultures *K. rhizophila* p3-3

**Fig. 3:**
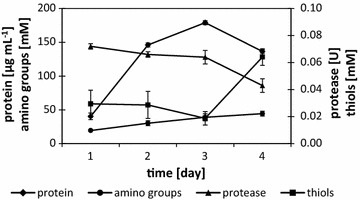
Culture course of *K. rhizophila* p3-3 in feather-containing medium. Proteolytic activity and accumulation of hydrolysis products were determined during culture of *K. rhizophila* p3-3 in the presence of 1% (w/v) feathers in agitated culture. Diamonds indicate protein concentration; circles indicate concentration of amino acids; triangles indicate proteolytic activity; squares indicate concentration of reduced thiols

Biodegradation course of feathers by *K. rhizophila* p3-3 was analyzed in 4-day submerged cultures in feather-containing medium, in terms of proteolytic activity and accumulation of hydrolysis products (Fig. 3). Highest production of proteases was observed on the initial day (0.072 U) of culture and was followed by a declining trend. The peak of soluble proteins released from the feather substrate appeared on the third day of culture and reached 179 μg/mL. The concentration of free amino groups was increasing throughout the tested culture course to reach a maximum value of 44.5 mM on the fourth day. The presence of reduced thiols in the growth environment was also confirmed.

**Fig. 4:**
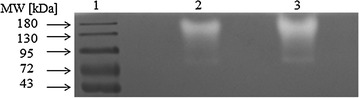
Casein zymography of proteases from *K. rhizophila* p3-3. The sample of culture fluid was taken from the 4-th day of culture in feather-containing medium. Lane 1—protein ladder; lane 2—5 μL sample; lane 3—10 μL sample

Zymographic analysis of the culture fluid was performed in polyacrylamide gel copolymerized with casein. Two activity bands were determined: a minor band of approx. 80 kDa and a dominating band between 130 and 180 kDa (Fig. 4).

**Fig. 5:**
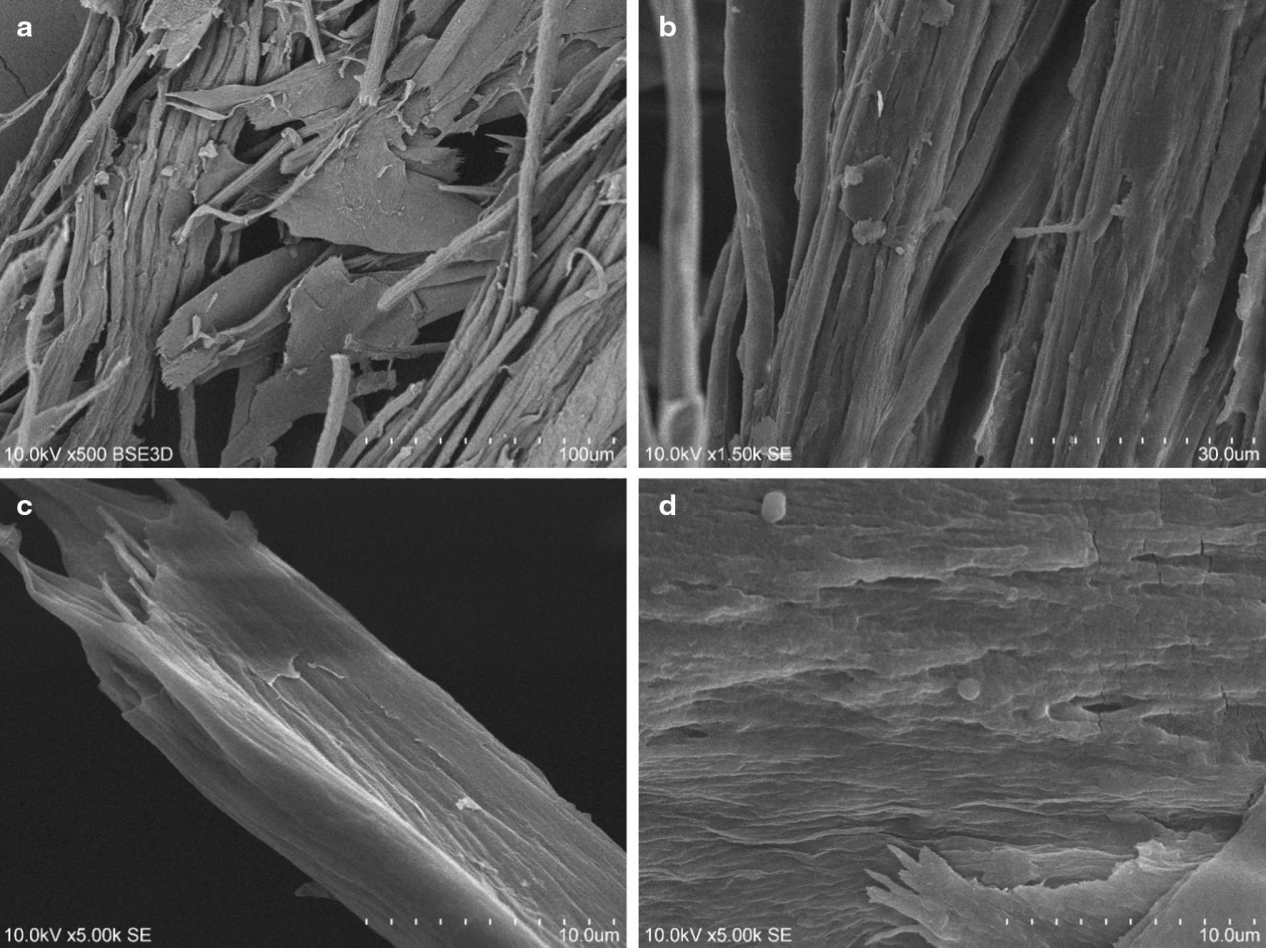
Scanning electron microscopy observations of feather degradation. SEM images of feather degradation after 4-day culture of *K. rhizophila* p3-3 depict: fragmentation of feather barbs (**a**), disruption of rachea (**b**), deterioration of barbule surface (**c**, **d**)

As a result of a degradative action of *K. rhizophila* p3-3 on the keratinous substrate, significant deterioration of feather structures was denoted. Detachment and advanced fragmentation of feather barbs, along with the disruption of the surface of rachea, were demonstrated in the SEM images (Fig. 5). Sparse colonization of the substrate surface by bacterial cells was observed.

### Effect of culture temperature on degradation of feathers

The process of feather biodegradation by *K. rhizophila* p3-3 was optimized. Determination of suitable culture temperature was performed, prior to the optimization procedure employing statistical models. It was verified, that most significant keratin biodegradation occurred in mesophilic conditions. Culture temperature of 25 °C allowed for both, highest substrate loss and maximum accumulation of hydrolysis products (Table 3). Increasing culture temperature by 5 °C resulted in virtually inhibited accumulation of proteins and amino acids, accompanied by a nearly 10% decreased substrate solubilization, despite comparable proteolytic activity. Additional temperature increment further diminished protease production and feather degradation.

**Table 3:** Effect of culturing temperature on feather substrate degradation

	Temperature			
	25 °C	30 °C	35 °C	40 °C
Proteins (µg/mL)	179 ± 2.8	143 ± 8.5	92 ± 7.1	140.5 ± 7.8
Amino groups (mM)	44.4 ± 3.5	16.1 ± 4.4	14.9 ± 3.9	30.1 ± 0.6
Proteolytic activity (U)	0.072 ± 0.014	0.070 ± 0.014	0.071 ± 0.005	0.047 ± 0.004
Substrate loss (% w/v)	51.9 ± 1.0	42.5 ± 1.6	36.4 ± 2.3	22.0 ± 0.1

### Screening of independent variables with Plackett–Burman design

The following step of the optimization was based on a Plackett–Burman experimental design, aimed at selection of culture parameters most influential for the release of proteins and amino acids from feathers during cultures of *K. rhizophila* p3-3.

The Plackett–Burman design is a useful and frequently applied tool for screening of independent variables that pose significant influence on the dependent variable. Nevertheless, its application requires certain discretion in drafting the intervals of tested parameters, as the model is strictly based on linear regression.

It was determined that all selected independent variables influenced the release of soluble proteins from the keratinous substrate (Table 4).

**Table 4:** Experimental layout and results of the Plackett–Burman experimental design

Run	X_1_	X_2_	X_3_	X_4_	X_5_	X_6_	X_7_	Protein (μg/mL)	Amino acids (mm)
1	− 1	− 1	− 1	+ 1	+ 1	+ 1	− 1	77.0 ± 4.2	33.37 ± 0.09
2	− 1	− 1	+ 1	+ 1	− 1	− 1	+ 1	116.5 ± 6.4	34.70 ± 0.28
3	− 1	+ 1	− 1	− 1	+ 1	− 1	+ 1	29.0 ± 11.3	20.45 ± 1.41
4	− 1	+ 1	+ 1	− 1	− 1	+ 1	− 1	42.5 ± 0.7	17.78 ± 1.04
5	+ 1	− 1	− 1	− 1	− 1	+ 1	+ 1	302.0 ± 18.4	62.08 ± 0.75
6	+ 1	− 1	+ 1	− 1	+ 1	− 1	− 1	453.5 ± 3.5	63.94 ± 3.20
7	+ 1	+ 1	− 1	+ 1	− 1	− 1	− 1	190.0 ± 4.2	60.88 ± 1.88
8	+ 1	+ 1	+ 1	+ 1	+ 1	+ 1	+ 1	242.5 ± 2.1	79.46 ± 1.04

**Fig. 6:**
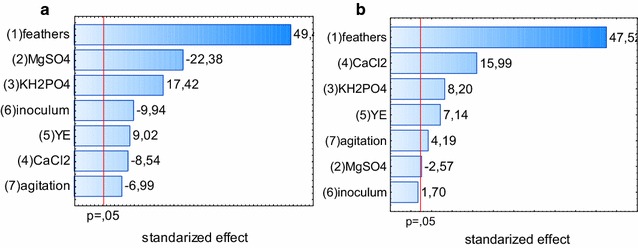
Pareto graph of effects. Pareto graph of standarized effects derived from the Plackett–Burman experimental design, concerning the release of soluble proteins (**a**) and amino acids (**b**)

As drawn from the Pareto graph of standardized effects, the highest influence was bound to the substrate concentration. Also, a negative effect of MgSO_4_ was observed, as well as a positive effect of KH_2_PO_4_ (Fig. 6a). The release of amino acids depended proportionally on the feather content and concentration of CaCl_2_ and phosphate, but not MgSO_4_ (Fig. 6b).

### Optimization of medium composition with a Box–Behnken design

The final stage of the optimization incorporated major influencing parameters, namely concentration of feathers, MgSO_4_ and KH_2_PO_4_, to define their effect on the release of soluble proteins from the keratinous substrate. Box–Behnken experimental design was applied to formulate the specific relationship between independent and dependent variables. The experiment was run according to the layout in Table 5.

**Table 5:** Experimental design with actual and predicted responses for the Box–Behnken design where the independent variables were designated: X_1_—feather content, X_2_—concentration of MgSO_4_, X_3_—concentration of KH_2_PO_4_

Run	X_1_	X_2_	X_3_	Actual response		Predicted response	
				ln protein (μg/mL)	Amino acid (mM)	ln protein (μg/mL)	Amino acid (mM)
1	− 1	− 1	0	5.453	45.29	5.389	37.86
2	+ 1	− 1	0	6.483	57.81	6.407	74.60
3	− 1	+ 1	0	4.934	47.69	5.010	30.90
4	+ 1	+ 1	0	5.926	166.51	5.990	173.94
5	− 1	0	− 1	5.198	43.83	5.124	68.52
6	+ 1	0	− 1	6.521	155.99	6.458	156.47
7	− 1	0	+ 1	5.414	48.75	5.477	48.27
8	+ 1	0	+ 1	6.065	164.78	6.140	140.09
9	0	− 1	− 1	6.070	112.83	6.209	95.56
10	0	+ 1	− 1	5.879	165.05	5.878	157.14
11	0	− 1	+ 1	6.292	84.72	6.294	92.63
12	0	+ 1	+ 1	5.966	106.17	5.827	123.43
13	0	0	0	5.793	157.72	5.727	148.93
14	0	0	0	5.677	167.31	5.727	148.93
15	0	0	0	5.771	133.74	5.727	148.93
16	0	0	0	5.666	136.94	5.727	148.93

Prior to the analysis, experimental data was evaluated for the need of transformation. The significant Chi2 of the Box–Cox transformation statistics with lambda of − 0.1467 suggested that residual sum of squares could be reduced (Additional file 2: Table S1). Therefore, a natural logarithm transformation was applied, which resulted in the insignificant Chi2 obtained, which suggested no need for further data transformation. Transformation of the dependent variable representing amino acid concentration was unnecessary, as the p value of Chi2 was below p = 0.05.

A regression model was developed for the process of microbial degradation of feathers, which was characterized by high suitability, as indicated by the high coefficient of determination R^2^ = 0.9683 (R^2^ adj. = 0.9206), which implied that over 96% of the variation of the dependent variable is described by the model. According to the model, significance of all three independent variables a was confirmed, however, the concentration of feather substrate (X_1_) affected positively protein release, while MgSO_4_ (X_2_) had a negative effect, however both exhibited linear influence on the response. The concentration of KH_2_PO_4_ represented a quadratic and negative effect (Table 6). In addition, significant interaction between variables X_1_ and X_3_ was shown.

**Table 6:** Effect evaluation of two regression models from the Box–Behnken design, for the release of soluble proteins and amino acids

Variable	Effect	Standard error	t value	p value
Release of soluble proteins				
Intercept	5.8502	0.0353	165.5901	< 0.0001
X_1_	0.9987	0.0865	11.5410	< 0.0001
X_2_	− 0.3983	0.0865	− 4.6020	0.0037
X_3_	0.0172	0.0865	0.1990	0.8488
X_1_X_1_	0.1400	0.0612	2.2878	0.0621
X_2_X_2_	− 0.1122	0.0612	− 1.8332	0.1165
X_3_X_3_	− 0.2128	0.0612	− 3.4768	0.0132
X_1_X_2_	− 0.0194	0.1224	− 0.1585	0.8792
X_1_X_3_	− 0.3359	0.1224	− 2.7446	0.0335
X_2_X_3_	− 0.0680	0.1224	− 0.5552	0.5988
Release of amino acids				
Intercept	99.9512	6.8766	14.5349	< 0.0001
X_1_	89.8828	16.8442	5.3361	0.0018
X_2_	46.1902	16.8442	2.7422	0.0336
X_3_	− 18.3162	16.8442	− 1.0874	0.3186
X_1_X_1_	41.7277	11.9107	3.5034	0.0128
X_2_X_2_	27.8740	11.9107	2.3403	0.0578
X_3_X_3_	3.8631	11.9107	0.3243	0.7567
X_1_X_2_	53.1504	23.8214	2.2312	0.0672
X_1_X_3_	1.9315	23.8214	0.0811	0.9380
X_2_X_3_	− 15.3856	23.8214	− 0.6459	0.5423

**Fig. 7:**
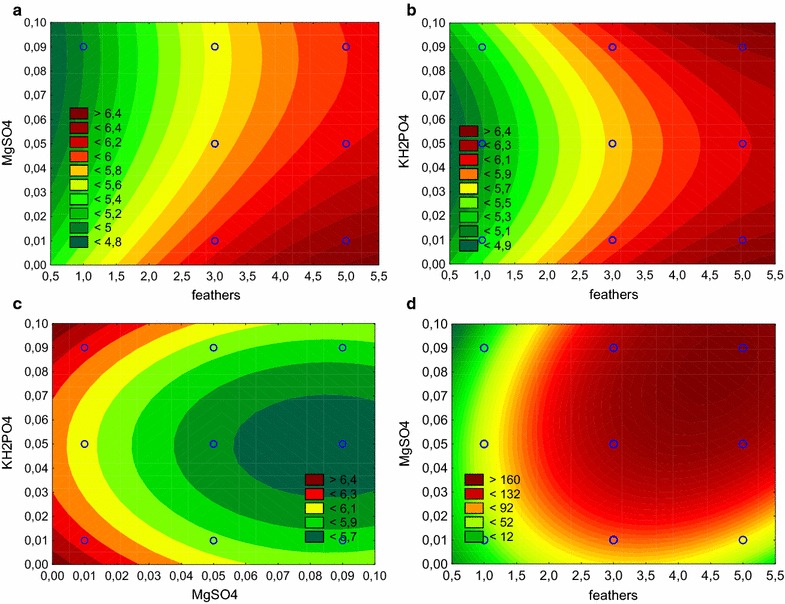
Response contour plots for the accumulation of soluble proteins (**a**–**c**) and amino acids (**d**) representing interaction effects of tested independent variables. Accumulation of soluble proteins as a function of feathers concentration vs MgSO_4_ (**a**), feathers vs KH_2_PO_4_ (**b**), MgSO_4_ vs KH_2_PO_4_ (**c**). Accumulation of free amino acids as a function of feathers concentration vs MgSO_4_ (**d**)

The analysis of standardized effects allowed to establish the following order of independent variables, according to their influence on the dependent variable: X_1_ > X_2_ > X_3_X_3_ > X_1_X_3_. ANOVA results for the obtained model inferred its significance, according to the F value of 20.3, additionally confirmed by the ‘‘lack of fit’’ tests of insignificant rank (Additional file 3: Table S2). The response surfaces were plotted to study interactions among tested factors. Linear characteristics of the variables X1 and X2 implied the shape of response surfaces with maximum values located at the edges of the plot (Fig. 7a, b). Hence, maximum applied concentration of substrate and minimum of MgSO_4_ resulted in maximum response. Non-linear effect of KH_2_PO_4_ combined with the interaction with the feather content resulted in a saddle characteristics, where the maximum applied concentration of both was preferential (Fig. 7c).

The obtained regression results allowed to define the polynomial equation (Eq. 2) describing the model (significant terms underlined):2

Previous screening of significant independent variables revealed that concentration of feathers, MgSO_4_ and KH_2_PO_4_ was also an influential factor for the release of amino acids in cultures of *K. rhizophila* p3-3, grown in feather medium (Table 6). Based on this fact, an additional regression model was developed, which was characterized by a good coefficient R^2^ = 0.9097 (R^2^ adj. = 0.7742) and acceptable F value = 6.7, followed by the “lack of fit” test with p = 0.1758 (Additional file 4: Table S3). When compared to the previously used Plackett–Burman model, it was confirmed that independent variables X_1_ and X_2_ were significant, however the variable X_3_, that represents the concentration of KH_2_PO_4_, did not produce a significant response.

The plotted response surface revealed a possible optimal point for the process of amino acids production from feathers, where the concentration of feathers and MgSO_4_ was at the level of 4.3 and 0.07%, respectively (Fig. 7d).

The regression results were used to define the polynomial equation (Eq. 3) to describe the model (significant terms underlined).3

### Evaluation of the feather hydrolysate

Finally, a concluding culture of *K. rhizophila* p3-3 was performed in the culture medium, in which three components were optimized. Specific concentrations of the components were selected, to achieve maximum concentration of soluble proteins, i.e. feathers 5.0%, MgSO_4_·7H_2_O 0.03%, KH_2_PO_4_ 0.01% (Additional file 5: Table S4). Concentration of remaining medium ingredients and culture parameters was taken from the performed Plackett–Burman model, depending on the positive or negative effect of either tested low (− 1) or high (+ 1) value. Maximum concentration of soluble proteins of 659 ± 34 µg/mL (678 µg/mL predicted) was attained on the third day of culture, with simultaneous 48.1 ± 1.5% loss of substrate weight.

Amino acid profile was determined in soluble fractions of feather hydrolysates directly in culture supernatant and after ultrasonic treatment or autoclaving of the raw culture broth. The treatments were aimed to enhance extraction of proteins and amino acids from bacterial cells. In the raw hydrolysate supernatant several dominating amino acids were determined, of which phenylalanine was dominant (approx. 50 μg/mL), as well as arginine, histidine, aspartic acid and alanine (Additional file 6: Table S5). The remaining amino acids were determined to appear below the level 10 μg/mL. The application of additional treatments to the culture broth allowed to increase the concentration of most amino acids in the supernatant by approximately 40% in total, however it did not affect the content of the prevailing phenylalanine.

In addition, anti-oxidative properties of the hydrolysate were evaluated using three analytical methods. Interesting free radical-scavenging potential was observed, mainly in relation to ABTS. Also, ferric reducing antioxidant power was determined. Additional treatments of the broth resulted in the increased anti-oxidative activities in the resultant hydrolysates (Table 7).

**Table 7:** Anti-oxidative properties of feather hydrolysates prior to and after treatments

	ABTS (μmol/mL)	DPPH (μmol/mL)	FRAP (mmol/mL)
Raw hydrolysate supernatant	5.34 ± 1.29	0.18 ± 0.08	0.60 ± 0.04
After ultrasound treatment	5.92 ± 0.96	0.27 ± 0.07	0.73 ± 0.04
After autoclaving	7.03 ± 1.34	0.24 ± 0.05	0.79 ± 0.05

## Discussion

One of the current trends in biotechnology is the application of microbial-mediated processes in valorization of food industry by-products, including keratinous wastes prom poultry processing. Exploitation of keratin proteins from poultry feather waste through enzymatic or microbial processes has been widely discussed in terms of prospects and economic conditions, where keratinolytic microorganisms often play a crucial role. From a total number of proteolytic bacterial isolates of poultry origin obtained in the study, nine exhibited considerable keratinolytic potential. Poultry plumage appeared to be a convenient source of keratinolytic microorganisms. Keratin-rich niches are typically considered as best isolation sites, that most include keratin waste dumps or living birds, however, some keratinolytic strains were acquired from other sources like soil or poultry farm sites. Nevertheless, the variety of the isolated bacteria did not reflect a typical composition of microflora in plumage, where occurrence of feather-degrading bacteria from the genera *Bacillus*, *Pseudomonas*, *Staphylococcus*, *Streptococcus*, *Stenotrophomonas* and *Escherichia* are most frequent (Shawkey et al. 2005; Sivakumar and Raveendran 2015). *Micrococcus* sp. or closely related *Kocuria* sp., although relatively abundant, are less frequently analyzed in terms of keratin-degrading capabilities, however if so, their present an immense potential. Thoroughly evaluated *Kocuria rosea* LPB-3, highly effective in biodegradation of chicken feathers was of soil origin (Vidal et al. 2000), while *M. luteus* also active on feathers was obtained from feather waste (Łaba et al. 2015).

A single isolate of *K. rhizophila* p3-3 that exhibited significant capabilities for feather degradation, was selected for the optimization study. The tested strain exhibited highest degradative capabilities at temperature 25 °C, in contrast to *K. rosea* cultured at 40 °C, as reported by Bernal et al. (2003). Despite that, final biodegradation rate of feathers was in high accordance between those two microorganisms.

Initially, typical medium was used, where besides basal components, feathers served as a main source of carbon and nitrogen. The medium was supplemented with yeast extract (0.5 g/L) which is often used to support initial growth of bacteria in the presence of a hardly degradable substrate (Barman et al. 2017). The selection was based on the maximum accumulation of keratin biodegradation products and proteolytic activity during growth in feather-containing medium. Concentration of free amino acids derived from decomposed keratin was superior in culture of *K. rhizophila* p3-3, as compared with cultures of *K. rosea*, capable of accumulating up to 26 mM amino acids (Vidal et al. 2000). Nevertheless, the dynamics of the process was comparable and represented a constantly growing trend. Concentration of soluble proteins was notable (179 μg/cm^3^) but it was lower than in cultures of highly proteolytic bacilli (de Oliveira et al. 2016). Reduced thiols were also detected in the culture fluid, however at the concentration below 0.1 mM. The presence of reduced cysteine residues is often considered as an indirect measure of keratin biodegradation, and is associated with proposed mechanisms of keratinolysis that involved synergistic action of enzymatic or chemical reducing factors (Korniłłowicz-Kowalska and Bohacz 2011). The result is in accordance with *M. luteus* B1pz, but in contrast to other microorganisms like *Bacillus* sp. or streptomyces, in cultures of which the concentration of free thiols could largely exceed 1 mM (Ramnani et al. 2005; Ramnani and Gupta 2007; Łaba et al. 2015).

Proteolytic activity *K. rhizophila* p3-3 in the unoptimized medium was below the level of 0.1 U, slightly lower in comparison proteases of *K. rosea*, recalculated from a comparable protocol, however in different conditions. Keratinolytic bacteria are typically associated with immense, at least one fold higher proteolytic activity against casein and a variety of proteinaceous substrates. Nevertheless, the undisputed feather-degrading capability of *K. rhizophila* might suggest the occurrence of complementary keratinolytic mechanisms.

The profile of proteolytic enzymes released into culture medium during growth on a proteinaceous substrate is a species-dependent feature and usually involves multiple activity bands present in zymograms. Proteolytic bacilli, which belong to the most frequently characterized keratinolytic bacteria, typically produce a number of activity bands, e.g. 7 in the case of *B. subtilis* 1271 (Mazotto et al. 2011) or 7 bands in cultures of *B. cereus* PCM 2849 (Łaba et al. 2017), both grown in feather-containing media. In contrast, keratinolytic cocci produced fewer extracellular proteases, i.e. two activity bands of > 200 kDa and a band of 90.2 kDa in culture of *K. rosea* (Bernal et al. 2003), or four proteases, > 200, 185, 139 and 62 kDa in cultures of *M. luteus* B1pz (Łaba et al. 2015).

Screening of culture parameters most influential for the accumulation of proteins and amino acids was conducted according to the Plackett–Burman design. The most influential parameter was concentration of the main substrate, namely feathers. It is natural that substrate concentration appears as one of the most important factors, not only for accumulation of degradation products, but also on keratinolytic activity (Paul et al. 2014). It determines not only carbon availability for bacteria and the initial output level of hydrolysis products, but also affects bacterial growth and enzyme activity through faster accumulation of products. The presence of additional carbon sources, like saccharides or peptones, besides the keratinous inducer, was confirmed by some authors to be relevant for production of keratinases (Ramnani and Gupta 2004; Cai and Zheng 2009), however it becomes less rational when biodegradation of the feather substrate is the goal. In the case of *K. rosea* feather substrate concentration along with magnesium sulphate appeared to be most influential for keratinase production (Bernal et al. 2006). Sulphates are typical mineral medium components for culturing bacteria in the presence of keratins, and is often considered in optimization studies. Nevertheless, the results from Plackett–Burman design revealed negative impact of increasing magnesium sulphate concentration in the tested range.

The change in concentration of yeast extract did not pose a significant effect on feather degradation in cultures of *K. rhizophila*, however, the role of this component varies for different bacteria. As an example, the addition of yeast extract is beneficial for both, proteolytic activity and biomass yield of *Micrococcus* sp. INIA 528 (Mohedano et al. 1997) and supports keratin biodegradation by *B. licheniformis* SHG10 (Embaby et al. 2015). Nevertheless, its excessive concentration could limit keratin biodegradation (Zaghloul et al. 2011).

The applied Box–Behnken design allowed to define relationships between three most influential medium components, feathers, MgSO_4_ and KH_2_PO_4_ that affect biodegradation of the feather substrate. The optimum raw feather content for keratinase production varies in different reports and concentrations below 1.5% are most frequent, however to maximize accumulation of hydrolysis products concentrations up to 8% were preferable (Embaby et al. 2010, 2015; Silva et al. 2014; Paul et al. 2014; Maciel et al. 2017). It is noteworthy that to maintain submerged cultivation maximum applicable concentration of raw down feathers is approximately 7% (w/v). In the presented study, concentration of proteins released from feathers almost linearly depended on their initial content, however, specific concentration of 4.25% was beneficial for increasing amino acids content. The negative influence on liberation of soluble proteins was in accordance with Plackett–Burman model, however it stimulated accumulation of amino acids in culture medium. The addition of 0.07% MgSO_4_ should be in regard if advanced keratin hydrolysis to amino acids is required. The saddle-type effect of KH_2_PO_4_ on the concentration of proteins implied that minimizing or even removal of this additional source of phosphorus should be considered.

The amino acid composition of the obtained hydrolysates soluble fraction, was a not only a result of the hydrolytic action of microbial enzymes on the keratinous substrate, but also of the enrichment of the hydrolysate with bacterial cell components. The predominant occurrence of glutamine and aspartic acid was in accordance with the feather hydrolysate produced by *K. rosea* (Bertsch and Coello 2005). However there were significant differences in the content of valine and leucine, typically most abundant in feather meals, but also histidine, methionine and phenylalanine (Adejumo et al. 2016). High concentration of the latter might be a result of the predominant chymotrypsin-like specificity of proteases, typical for many known keratinases (Brandelli et al. 2010). Nonetheless, according to Bertsch and Coello (2005), fermentation of feathers within a culture of *K. rosea* was advantageous in order to improve the amino acid balance of the keratin hydrolysate, but also to improve the overall digestibility of the product.

It is notable, that feather hydrolysates obtained during fermentation with *K. rhizophila* p3-3 exhibited significant free radical-scavenging activity, as well as ferric reducing antioxidant power. Antioxidative properties of protein hydrolysates of plant and animal origin, including feather and wool hydrolysates, are recently of special interest. This antioxidative potential is known to occur due to the presence of bioactive peptides, which in turn, are dependent on enzymes specificity and a substrate applied in the hydrolysis.

## Additional files

**Additional file 1: Figure S1.** Screening of bacterial isolates for proteolytic activity. Proteolytic activity was determined on skim milk agar plates and expressed as clear zone diameter. Isolates obtained from feather samples were designated with letter “p”; grey bars indicate isolates selected for further study.

**Additional file 2: Table S1.** Box–Cox transformation statistics of dependent variables.

**Additional file 3: Table S2.** Analysis of variance (ANOVA) for the obtained regression model for the release of soluble proteins.

**Additional file 4: Table S3.** Analysis of variance (ANOVA) for the obtained regression model for the release of amino acids.

**Additional file 5: Table S4.** Determined values of independent variables to maximize different responses.

**Additional file 6: Table S5.** Concentration of dominant amino acids in feather hydrolysates prior to and after treatments.

## Abbreviations

LB: lysogeny broth
FM: feather medium
rpm: revolutions per minute
PCR: polymerase chain reaction
RDP: Ribosomal Database Project
MAFFT: multiple alignment using fast Fourier transform
Tris: tris(hydroxymethyl)aminomethane
TCA: trichloroacetic acid
ABTS: 2,2′-azino-bis(3-ethylbenzthiazoline-6-sulfonic acid)
DPPH: 2,2-diphenyl-1-picrylhydrazyl
FRAP: ferric reducing antioxidant power
SEM: scanning electron microscopy
HPLC: high-performance liquid chromatography
ANOVA: analysis of variance
