# Supplementary material for: New keratinolytic bacteria in valorization of chicken feather waste
Source: AMB Express. 2018 Jan 24;8:9. doi: 10.1186/s13568-018-0538-y (PMC5783986; doi:10.1186/s13568-018-0538-y)
Supplement: Supplementary file 2 — Additional file 2: Table S1. Box–Cox transformation statistics of dependent variables. [file 13568_2018_538_MOESM2_ESM.docx]

| dependent variable | lambda | MS | SSE | Chi^2^(1) | p-value |
| --- | --- | --- | --- | --- | --- |
| soluble proteins | -0.1467 | 3200.01 | 10034.7 | 10.3819 | 0.0013 |
| soluble proteins (ln transformed) | 0.2579 | 0.0150 | 0.0888 | 0.1963 | 0.6577 |
| amino acids | -0.0771 | 567.46 | 2860.0 | 2.7898 | 0.0949 |

Table S1. Box-Cox transformation statistics of dependent variables
