# Supplementary material for: New keratinolytic bacteria in valorization of chicken feather waste
Source: AMB Express. 2018 Jan 24;8:9. doi: 10.1186/s13568-018-0538-y (PMC5783986; doi:10.1186/s13568-018-0538-y)
Supplement: Supplementary file 3 — Additional file 3: Table S2. Analysis of variance (ANOVA) for the obtained regression model for the release of soluble proteins. [file 13568_2018_538_MOESM3_ESM.docx]

| source | sum of squares (SS) | degrees of freedom (DF) | mean square (MS) | F-value | p-value |
| --- | --- | --- | --- | --- | --- |
| regression model | 2.7405 | 9 | 0.3045 | 20.3 | <0.001 |
| residual error | 0.0898 | 6 | 0.0150 | - | - |
| lack of fit | 0.0773 | 3 | 0.0258 | 6.1742 | 0.0846 |
| pure error | 0.0125 | 3 | 0.0042 | - | - |
| cor. total | 2.8303 | 15 | - | - | - |

Table S2. Analysis of variance (ANOVA) for the obtained regression model for the release of soluble proteins

R^2^= 0.9683; R^2^ adj. = 0.9206
