# Supplementary material for: New keratinolytic bacteria in valorization of chicken feather waste
Source: AMB Express. 2018 Jan 24;8:9. doi: 10.1186/s13568-018-0538-y (PMC5783986; doi:10.1186/s13568-018-0538-y)
Supplement: Supplementary file 4 — Additional file 4: Table S3. Analysis of variance (ANOVA) for the obtained regression model for the release of amino acids. [file 13568_2018_538_MOESM4_ESM.docx]

| Source | sum of squares (SS) | degrees of freedom (DF) | mean square (MS) | F-value | p-value |
| --- | --- | --- | --- | --- | --- |
| regression model | 34293.62 | 9 | 3810.40 | 6.7 | <0.025 |
| residual error | 3404.74 | 6 | 567.46 | - | - |
| lack of fit | 2615.17 | 3 | 871.72 | 3.3121 | 0.1758 |
| pure error | 789.57 | 3 | 263.19 | - | - |
| cor. Total | 37698.36 | 15 | - | - | - |

Table S3. Analysis of variance (ANOVA) for the obtained regression model for the release of amino acids

R^2^= 0.9097; R^2^ adj. = 0.7742
