# Supplementary material for: New keratinolytic bacteria in valorization of chicken feather waste
Source: AMB Express. 2018 Jan 24;8:9. doi: 10.1186/s13568-018-0538-y (PMC5783986; doi:10.1186/s13568-018-0538-y)
Supplement: Supplementary file 5 — Additional file 5: Table S4. Determined values of independent variables to maximize different responses. [file 13568_2018_538_MOESM5_ESM.docx]

Table S4. Determined values of independent variables to maximize different responses

| optimized response | feathers | MgSO_4_∙7H_2_O | KH_2_PO_4_ |
| --- | --- | --- | --- |
| protein | 5.0% | 0.03% | 0.01% |
| amino acids | 4.3% | 0.07% | 0.03% |
| protein + amino acids | 5.0% | 0.06% | 0.01% |
