# Supplementary material for: New keratinolytic bacteria in valorization of chicken feather waste
Source: AMB Express. 2018 Jan 24;8:9. doi: 10.1186/s13568-018-0538-y (PMC5783986; doi:10.1186/s13568-018-0538-y)
Supplement: Supplementary file 6 — Additional file 6: Table S5. Concentration of dominant amino acids in feather hydrolysates prior to and after treatments. [file 13568_2018_538_MOESM6_ESM.docx]

Table S5. Concentration of dominant amino acids in feather hydrolysates prior to and after treatments

| amino acid | concentration [μg/mL] | | |
| --- | --- | --- | --- |
|  | raw hydrolysate supernatant | after ultrasound treatment | after autoclaving |
| Asp | 24.4 | 26.6 | 37.8 |
| Glu | bd | 32.6 | 52.4 |
| Ser | 6.4 | 7.8 | 7.4 |
| Gln | bd | 14.9 | 12.5 |
| His | 38.2 | 50.9 | 61.4 |
| Ala | 16.5 | 19.8 | 26.1 |
| Arg | 29.2 | 33.5 | 38.9 |
| Tyr | bd | 2.2 | 3.1 |
| Met | 9.3 | 10.4 | 11.2 |
| Phe | 50.7 | 50.4 | 50.5 |
| Ile | 4.7 | 7.5 | 5.9 |
| Leu | 2.1 | 4.7 | 4.2 |
| Lys | 5.9 | 6.8 | 6.0 |

bd – below detection limit
