# Supplementary material for: New keratinolytic bacteria in valorization of chicken feather waste
Source: AMB Express. 2018 Jan 24;8:9. doi: 10.1186/s13568-018-0538-y (PMC5783986; doi:10.1186/s13568-018-0538-y)
Supplement: Supplementary file 6 [file 13568_2018_538_MOESM1_ESM.docx]

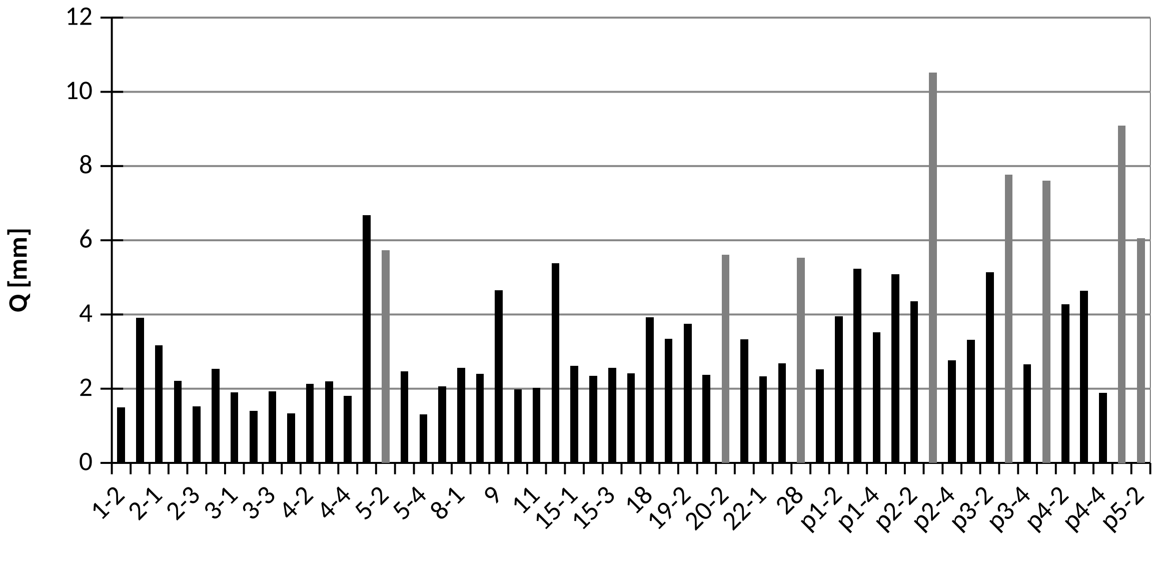

Q [mm]
0
2
4
6
8
10
12
1-2
2-1
2-3
3-1
3-3
4-2
4-4
5-2
5-4
8-1
9
11
15-1
15-3
18
19-2
20-2
22-1
28
p1-2
p1-4
p2-2
p2-4
p3-2
p3-4
p4-2
p4-4
p5-2
